# The Role of Plzf in Spermatogonial Stem Cell Maintenance and Differentiation: Mapping the Transcriptional Dynamics and Key Interactions

**DOI:** 10.3390/cells13231930

**Published:** 2024-11-21

**Authors:** Nima Ghasemi, Hossein Azizi, Seyedeh-Kiana Razavi-Amoli, Thomas Skutella

**Affiliations:** 1 Faculty of Biotechnology, Amol University of Special Modern Technologies, P.O. Box 49767, Amol 4615664616, Iran; nima.ghasemi@ausmt.ac.ir; 2 Student Research Committee, School of Medicine, Mazandaran University of Medical Sciences, Sari 4815733971, Iran; kianarazavi2016@gmail.com; 3 Institute for Anatomy and Cell Biology, Medical Faculty, University of Heidelberg, Im Neuenheimer Feld 307, 69120 Heidelberg, Germany; thomas.skutella@uni-heidelberg.de

**Keywords:** Spermatogonial stem cells (SSCs), Promyelocytic leukemia zinc-finger (PLZF), Spermatogenesis, Immunostaining, Bioinformatics analysis

## Abstract

Spermatogonial stem cells (SSCs) sustain and modulate spermatogenesis through intricate signaling pathways and transcription factors. Promyelocytic leukemia zinc-finger (*Plzf*, also known as *Zbtb16*) has been identified as a critical transcription factor influencing various signaling and differentiation pathways. *Plzf* plays a pivotal role in regulating the differentiation properties of SSCs and is essential for the proper maintenance of spermatogenesis. However, the transcription patterns of *Plzf* along the seminiferous tubules and its interaction network with adjacent partners still need to be fully elucidated. This study employed immunostaining techniques coupled with Fluidigm quantitative real-time polymerase chain reaction (Fluidigm qPCR) to quantify *Plzf* expression in undifferentiated and differentiated spermatogonia. Furthermore, we utilized bioinformatics analyses to identify *Plzf* partners and their associations with other regulatory factors. Immunohistostaining (IMH) revealed a high expression of *Plzf* in cells near the basal membrane of seminiferous tubules and a lower expression in the middle regions in vivo. Immunocytochemistry (ICC) demonstrated that undifferentiated spermatogonia exhibited significant *Plzf* positivity, whereas differentiated spermatogonia showed reduced *Plzf* expression in vitro. Fluidigm qPCR confirmed a significant differential expression of *Plzf* between undifferentiated and differentiated spermatogonia. In silico differential expression analysis between undifferentiated spermatogonia and spermatids indicated that *Plzf* is closely associated with *Mycn*, *Lin28a*, *Kras*, *Ccnd1*, and *Jak1*, highlighting the importance of these partnerships during spermatogenesis. Our findings suggest that the network of *Plzf*-related partners and their associated proteins involves differentiation, localization, apoptosis, and signal transduction. This comprehensive approach advances our understanding of *Plzf* transcription patterns and sheds light on its interactions with other cellular factors, revealing previously obscure pathways and interactions. These insights could lead to more effective diagnostic strategies for reproductive system-related diseases and inform the development of improved therapeutic and clinical applications.

## 1. Introduction

Spermatogonial stem cells (SSCs) are responsible for spermatogenesis, a crucial process in the male reproductive system. SSCs have two main functions. They maintain the stem cell pool by the self-renewal capacity through the miotic divisions and also drive spermatogenesis by differentiating from undifferentiated to differentiated spermatogonia [1]. SSCs are present in the basement membrane of seminiferous tubules and receive support from Sertoli cells, peritubular myoid cells, and other somatic cells. These somatic cells help determine the fate of SSCs through extracellular signals and growth factor secretion [2]. In mouse spermatogenesis, spermatogonia undergo several stages of differentiation to form spermatozoa. Based on their topographical organization, undifferentiated spermatogonia are categorized as single A (As), paired A (Apr), or aligned A (Aal) cells [1]. Within this undifferentiated group, SSCs are a subset of As cells that maintain the stem cell pool through self-renewal. Most As, Apr, and Aal cells eventually differentiate into A1 spermatogonia, marking the transition to progenitor cells. Once committed, these cells undergo a series of divisions (A1 to A2, eventually reaching the B spermatogonia stage) and produce primary and secondary spermatocytes. Through further divisions, secondary spermatocytes transform into round spermatids and ultimately into spermatozoa [3]. Collectively, the A1 to B spermatogonia are referred to as differentiated spermatogonia [4].

Spermatogenesis is a complex, highly regulated developmental process influenced by factors such as steroid hormones (including androgens and estrogens), which are essential for initiating and sustaining this process. These sex hormones exert their influence by different mechanism routes [5]. They can act as the direct activator of genes by binding to hormone-dependent promoter elements [6]. Steroid hormones can modulate gene expression by activating the hormone-dependent transcription factors, leading to secondary transcriptional responses [6,7]. Additionally, steroid hormones also regulate spermatogenesis by inducing transcription-independent events, often called nonclassical pathways [8].

In addition, and in interaction with hormones, mainly transcription factors regulate spermatogenesis. Diverse families of transcription factors are present in this process, including homeobox transcription factors, zinc-finger transcription factors, heat-shock transcription factors, and cAMP-response transcription factors. These factors, along with epigenetic modulations such as DNA methylation and post-translational modification regulation, are some of the most crucial spermatogenesis-affecting factors [9,10].

Promyelocytic leukemia zinc finger (*Plzf*), also known as *ZBTB16*, is a transcription factor essential for various cellular processes, including differentiation and self-renewal. Additionally, it has been demonstrated that PLZF-induced growth suppression and apoptosis in Jurkat cells [11]. It belongs to the BTB/POZ-zinc finger family of proteins, known for their roles in gene repression and chromatin remodeling [9,12]. *Plzf* is particularly significant in the context of spermatogenesis. *Plzf* is crucial for the continuous production of sperm throughout spermatogenesis. During spermatogenesis, *Plzf* is predominantly expressed in undifferentiated spermatogonia, which maintains the balance between self-renewal and differentiation of spermatogonia. *Plzf* regulates the transcription of target genes involved in cell cycle control and differentiation by binding to specific DNA sequences [9]. In the context of spermatogenesis, *Plzf* represses genes that promote differentiation, and, as a result, maintains the spermatogonial stem cell population in an undifferentiated state. This guarantees the spermatogenesis continuance over time by preserving the SSC population [13]. *Plzf* gene knockout has been shown to have significant effects on spermatogenesis. For example, studies using knockout mouse models have demonstrated that the absence of *Plzf* leads to a depletion in undifferentiated spermatogonia, resulting in impaired spermatogenesis and infertility [9]. Moreover, *Plzf* protects SSCs from genotoxic stress, ensuring their genomic integrity through successive rounds of cell division by influencing a network of downstream effectors involved in this process [14,15].

Identifying and characterizing the upstream signaling pathways regulating *Plzf* activity in spermatogonial stem cells (SSCs) is crucial. Understanding these pathways could reveal how *Plzf* activity is modulated in response to different physiological conditions. Although some target genes of *Plzf* have been identified, a comprehensive understanding of the *Plzf*-regulated gene network still needs to be provided. Functional characterization of these target genes, including their roles in SSC maintenance and differentiation, and detailed knowledge of PLZF’s protein–protein interactions and cross-talk with other signaling pathways is essential. As a result, understanding the role of *Plzf* in SSCs could inform strategies for stem cell-based therapies in treating infertility. Furthermore, identifying small molecules or other therapeutic agents that can modulate *Plzf* or its close partner’s activity might offer new treatments related to SSC dysfunction.

In this research, we hypothesized the possible role of Plzf as a critical factor in maintaining the balance between self-renewal and differentiation of SSCs, which is essential for continuous spermatogenesis in males. We aim to investigate the changes in Plzf expression across the seminiferous epithelium and during different stages of SSC maturation. Through our experiments, including immunohistochemistry, immunocytochemistry, qPCR, microarray analysis, and protein–protein interaction network construction, the study seeks to understand the Plzf-regulated gene network and its interactions with other signaling pathways. Ultimately, we aim to identify upstream signaling pathways that regulate Plzf activity and explore its potential as a therapeutic target for treating infertility and SSC-related disorders. Based on in vivo immunostaining experiments, Plzf is transcribed in the basal region of the seminiferous tubules in cells known as undifferentiated spermatogonia. However, Plzf expression is notably low or absent in other seminiferous tubular regions, particularly in differentiated spermatogonia and precursor germ cells. Immunocytochemistry further revealed a significant difference in Plzf gene expression between differentiated and undifferentiated spermatogonia, showing the possibility of Plzf’s role in maintaining the undifferentiated state. Microarray analysis identified Plzf as a critical gene in spermatogenesis. Constructing a PPI network based on microarray results revealed a close association between PLZF and other significant regulators such as nMYC, LIN28A, FOXO1, and JAK1. Enrichment analysis of the PLZF-associated cluster demonstrated that PLZF and its interacting partners are involved in “cellular localization”, “regulation of the MAPK cascade”, “cell differentiation”, and “apoptosis”.

*Plzf* may play a critical role in maintaining the balance between self-renewal and differentiation of SSCs, essential for continuous spermatogenesis in a male’s life. Its expression is predominantly observed in undifferentiated spermatogonia, with significantly lower levels in differentiated spermatogonia, underscoring its importance in preserving the undifferentiated state. It closely interacts with other key proteins involved in spermatogenesis and stem cell controllers. *Plzf* closely interacts with other key proteins involved in spermatogenesis and stem cell regulation. Future research should focus on identifying the upstream signaling pathways regulating *Plzf* activity and exploring its potential as a therapeutic target for treating infertility and SSC-related disorders by modulating its function or interacting partners.

## 2. Material and Methods

### 2.1. Testis Digestion and Culture of Testicular Cells

Testicular cells were isolated from C57BL/6 mouse strains using GFP transgenic mice with an Oct4-promoter reporter system. The cells underwent decapsulation followed by a one-step enzymatic digestion process. The digestion, suspension, and plating procedures were carried out as described in our previous work [16]. The cells were then cultured in mouse GSC (mGSC) medium and maintained in a 37 °C environment with 5% CO_2_, following the protocol established in our earlier studies [16].

### 2.2. Electron Microscopy

For electron microscopy, undifferentiated and differentiating spermatogonia were initially pelleted and fixed in a paraformaldehyde/glutaraldehyde solution buffered with PIPES. The fixative was refreshed after a 5 min preliminary fixation to ensure optimal preservation. The pellets were then post-fixed in a solution of osmium tetroxide (OsO4) and potassium hexacyanoferrate for 50 min, followed by rinsing in a uranyl acetate buffer and a sodium maleate buffer at pH 6.0. The samples were block-stained with uranyl acetate. The tissue was dehydrated through a graded ethanol series, starting with brief 5 min treatments and four extended 20 min steps. The samples were then embedded in Epon resin and polymerized at 60 °C for 24 h. Ultrathin sections, approximately 50 nm thick, were prepared and visualized using a Zeiss EM10 electron microscope (Carl Zeiss, Oberkochen, Germany).

### 2.3. Tissue Processing and Immunostaining Steps

Testicular tissue was meticulously harvested from male animals, followed by thorough washing with PBS and fixation in a 4% paraformaldehyde solution to preserve cellular morphology. The comprehensive procedures for tissue preparation, embedding, sectioning, and staining were conducted by the established protocols outlined in our previous work [16]. Before the immunostaining, the frozen sections were allowed to air-dry at room temperature for 30 min to facilitate optimal adhesion. For immunostaining, cultured cells were maintained in 24-well plates and subsequently fixed with 4% paraformaldehyde. After washing with PBS, the samples underwent permeabilization using 0.1% Triton in PBS, followed by blocking with 1% BSA in PBS to minimize non-specific binding. The blocking solution was then discarded, and the cells were incubated overnight with primary antibodies to ensure thorough binding. After further washing, the cells were treated with species-specific secondary antibodies conjugated to various fluorochromes for enhanced visualization. Subsequently, the cells were counterstained with 0.2 μg/mL DAPI (4′,6-diamidino-2-phenylindole) for 3 min at room temperature, then fixation with Mowiol 4-88 reagent. The labeled cells were meticulously analyzed using a Zeiss LSM 700 confocal microscope (Carl Zeiss Microscopy, Jena, Germany)., and high-resolution images were captured with a Zeiss LSM-TPMT camera (Carl Zeiss, Jena, Germany), allowing for detailed examination of cellular structures.

### 2.4. Gene Expression Analyses on the Fluidigm Biomark System

The *Plzf* and *Vasa* gene expression in adult testicular cells was measured using Fluidigm dynamic array chips. *GAPDH* served as the housekeeping gene for normalization. Selected cultured testicular cells were lysed in a lysis buffer containing 1.3 μL TE buffer, 9 μL RT-PreAmp Master Mix, 0.2 μL R.T./Taq Superscript III (Invitrogen, Waltham, MA, USA), 2.5 μL 0.2× assay pool, and 5.0 μL Cells Direct 2× Reaction Mix (Invitrogen, Waltham, MA, USA). Targeted transcripts were quantified with TaqMan real-time PCR on the Biomark real-time quantitative PCR system. The qPCR analysis was conducted with four biological replicates, ensuring the results reflect biological variability. Additionally, each sample was tested in two technical replicates to account for experimental precision and reduce potential variability introduced during the qPCR process. Ct values were analyzed using GenEx software (version 5.4.2, MultiD Analyses, Gothenburg, Sweden).

### 2.5. Microarray Data Analysis and Data Normalization

This study utilized dataset GSE57197, which includes 23 samples, obtained from the NCBI GEO database, for microarray analysis. From this dataset, four spermatogonia samples and four round spermatid samples were selected [17]. Analysis was conducted using the GPL1261 (Mouse430_2) Affymetrix Mouse Genome 430 2.0 Array platform. Expression files were analyzed using the Transcriptome Analysis Console (TAC) software (version 4.0). The RMA normalization method and the ANOVA method were applied. Genes with an FDR-corrected *p*-value less than 0.05 and a fold change less than −2 or greater than 2 were identified as significantly differentially expressed genes (DEGs).

### 2.6. Protein–Protein Interaction (PPI) Networks Construction and Modularity Analysis

In this research, STRING (v.12.0) (https://string-db.org/) (accessed on 31 July 2024) was used to predict protein–protein interactions (PPI) among the differentially expressed genes (DEGs) identified. The analysis was performed for *Mus musculus* with a medium confidence score of 0.400. The PPI network was imported into Cytoscape (v.3.6.0) and Centiscape plugin (v.2.2) in Cytoscape for additional analysis. Moreover, protein functional clusters (modules) were determined using the modularity function in the Gephi app (v.0.10.1).

### 2.7. Enrichment Analysis

To gain a deeper insight into the functional clusters (modules) of each protein and the DEGs within the network, enrichment analysis was conducted using the enrichment analysis tool of the STRING plugin (v. 2.1.1) of the Cytoscape app. The enrichment results were sourced from KEGG, Wikipathways, Reactome databases, and Gene Ontology (GO) terms (accessed on 10 August 2024).

### 2.8. AI Tools

Artificial intelligence tools are strictly forbidden for tasks such as data generation, article structuring, scientific conclusion drawing, or any actions that might lead to scientific misrepresentation. These tools are intended solely for purposes of text editing and grammar correction. Their application is confined to improving the clarity and precision of the content, thereby ensuring that there is no alteration or misrepresentation of scientific data within the discussions.

### 2.9. Statistical Analysis

The experiments were conducted four times to ensure the reliability of the findings. The collected data were thoroughly analyzed using the Statistical Package for the Social Sciences (SPSS) version 27.0. The Shapiro–Wilk test was utilized to evaluate the normality of gene expression data obtained from the Fluidigm qPCR. A *p*-value exceeding 0.05 indicated that the data followed a normal distribution, thereby permitting the use of parametric tests, such as one-way analysis of variance (ANOVA). In contrast, a *p*-value below 0.05 suggested a non-normal distribution, leading to the use of non-parametric tests, such as the Kruskal–Wallis H test. The Bonferroni post hoc test was employed to assess the significance of differences between groups, with statistical significance set at a *p*-value of less than 0.05.

## 3. Results

### 3.1. In Vivo Gene Expression Across Seminiferous Tubules by Immunohistochemistry Test

**Figure 1:**
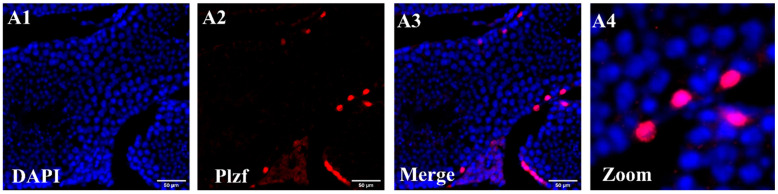
(**A1**–**A4**) The immunohistochemistry image of seminiferous tubules stained for PLZF reveals a distinct population of cells located near the basal membrane—identified as undifferentiated spermatogonia—that are positive for PLZF. In contrast, other cell types within the tubules do not express Plzf. (Scale bar = 50 µm).

We analyzed *Plzf* expression in tubules-mouse seminiferous tubular sections using immunohistochemistry (IMH). The IMH analysis revealed a high nuclear expression of Plzf in specific basal compartment cells, which can be identified as entirely undifferentiated spermatogonia. Notably, undifferentiated spermatogonia are the only cells within the seminiferous tubules that show positive PLZF staining. In contrast, other cell types located near the basal membrane, within the middle compartment, and near the luminal region of the seminiferous tubules—such as Sertoli cells, partially differentiated germ cells, precursor cells, differentiating SSCs and spermatids—are negative for *Plzf* expression (Figure 1). This aligns with our reanalysis of the publicly available dataset GSE57197, which revealed a significant negative fold change in *Plzf* expression in spermatids compared to undifferentiated spermatogonia.

**Figure 2:**
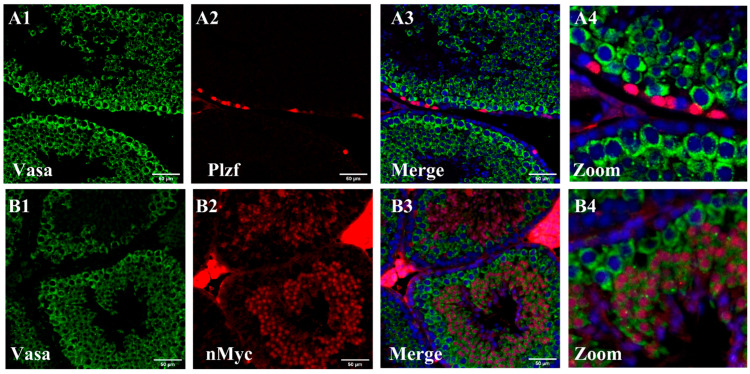
Immunohistochemistry (IHC) analysis of testis cross-sections reveals that VASA-positive cells do not express *Plzf* (**A1**–**A4**). Among the VASA-positive population, a subset shows positivity for nMYC. In contrast, another group of VASA-positive cells located near the basal membrane of the seminiferous tubules remains negative for nMYC (**B1**–**B4**). In contrast, the more differentiated cell tubules near the tubules’ luminal region with more advanced differentiation status are negative for VASA, NMYC, and PLZF. Partial tubule images were used to highlight specific structures and the intense staining in the interstitial regions is likely due to autofluorescence (Scale bar = 50 µm).

Additionally, we assessed the protein levels of nMYC and VASA, which are recognized as key regulators of spermatogenesis, through double-staining IMH with PLZF. The results showed that PLZF-positive fully undifferentiated spermatogonia exhibit low expression of VASA protein. However, the middle compartment of the seminiferous tubules, which contains part of undifferentiated spermatogonia, precursor cells, and differentiated spermatogonia, shows cytoplasmic expression of *Vasa*. Also, nMYC protein expression was not significant near the basal membrane of the seminiferous tubules in differentiated spermatogonia. However, nMYC exhibits nuclear expression in the middle compartment, containing precursor cells. These findings suggest that *Plzf* is prominently expressed in undifferentiated spermatogonia, whereas *nMyc* and *Vasa* are minimally expressed in these cells in vivo. Conversely, nMYC and VASA-positive cells, located in the middle compartment of the seminiferous tubules and likely representing differentiating spermatogonia, are negative for PLZF. Interestingly, not all VASA-positive cells are positive for nMYC. Furthermore, cells located near the luminal region of the seminiferous tubules are negative for nMYC, VASA, and PLZF (Figure 2). This observation suggests that as spermatogenesis progresses, the expression of these three genes is downregulated in the later stages of the process, particularly in cells predicted to be spermatids. The low or absent *nMyc* expression in the luminal regions of the seminiferous tubules in cells presumed to be spermatids is consistent with our microarray analysis, which showed a significant negative fold change in *nMyc* expression during differentiation into spermatids. However, comparing immunohistostaining with microarray analysis does not allow us to assess *nMyc* expression in undifferentiated spermatogonia near the basal membrane.

### 3.2. In Vitro Gene Expression of Spermatogonial Stem Cells by Immunocytochemistry Test

**Figure 3:**
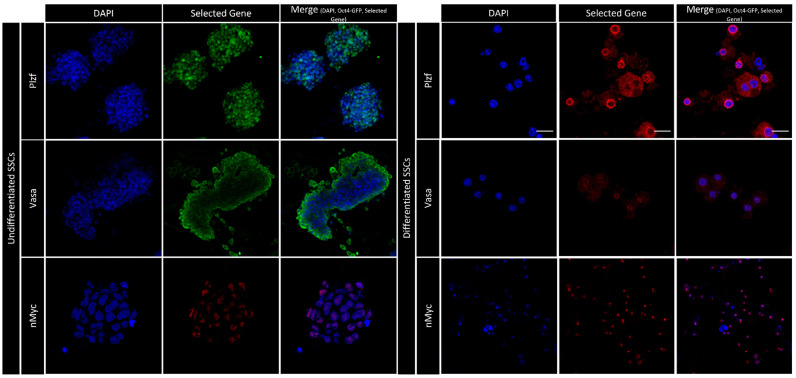
Immunocytochemistry (ICC) reveals the expression of *Plzf*, *Vasa*, and *nMyc* (green or red) with DAPI nuclear counterstain (blue) in undifferentiated (left panel) and differentiated (right panel) spermatogonia. Plzf and Vasa show robust expression in undifferentiated spermatogonia, significantly diminishing upon differentiation. nMYC, on the other hand, demonstrates prominent nuclear localization in differentiated spermatogonia, while its nuclear expression remains relatively low in undifferentiating spermatogonia. The merged images display the colocalization of these gene markers with nuclear staining. (Scale bar = 50 µm (All images except nMYC in differentiated spermatogonia (Scale bar = 100 µm)).

Following enzyme digestion, the isolated cells were cultured with growth factors. The characterization of these testicular cells was carried out in detail in our earlier [18]. *Plzf* gene expression was assessed using immunocytochemistry (ICC) for undifferentiated and differentiated spermatogonia in vitro. The ICC results demonstrated that undifferentiated spermatogonia exhibit high levels of *Plzf* expression, while differentiated spermatogonia show minimal cytoplasmic *Plzf* expression. These findings are consistent with our immunohistochemistry (IMH) results to some extent. Additionally, our data indicate that *nMyc* is expressed at low levels in undifferentiated spermatogonia but is more prominent in differentiated spermatogonia (Figure 3). In contrast, while the IMH results showed that undifferentiated spermatogonia have a low expression of *Vasa*, the ICC data revealed that these cells express *Vasa* as like as differentiating spermatogonia. This controversy might be because of in vitro conditions such as cells removal from their natural niche, absence of Sertoli cells, tissue-specific molecules, and cytokines, potentially affecting their gene expression profiles potentially affecting their gene expression profiles.

### 3.3. Analyses of Genes Expression by Fluidigm Biomark System Between Spermatogonial Stem Cells

**Figure 4:**
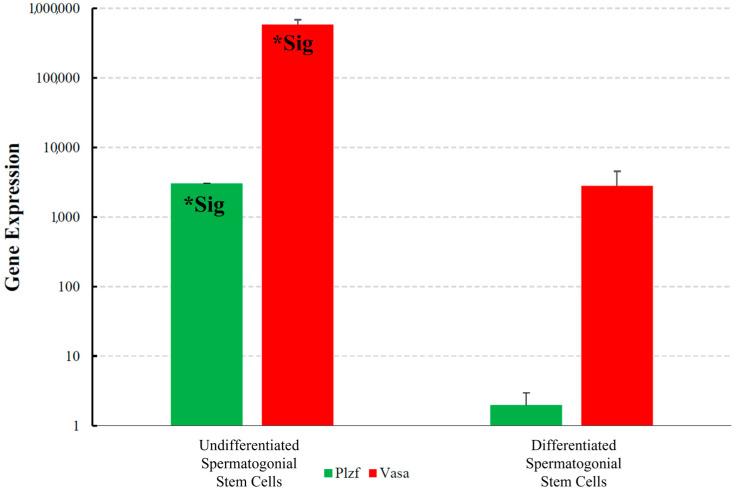
The results of the Fluidigm qPCR analysis display the fold change in mRNA expression (*Y*-axis) on a logarithmic scale (Base 10). The “Sig*” symbol indicates a statistically significant difference in gene expression (*p* < 0.05) for the analyzed genes. The analysis revealed significantly different mRNA expression levels of *Vasa* and *Plzf* in undifferentiated spermatogonia compared to differentiating, underscoring their roles in spermatogenesis.

Fluidigm qPCR showed a significant expression of *Plzf* and *Vasa* in differentiating spermatogonia compared to undifferentiated spermatogonia (Figure 4).

### 3.4. Microarray Analysis and Identification of Differentially Expressed Genes in the Spermatogenesis Process

**Figure 5:**
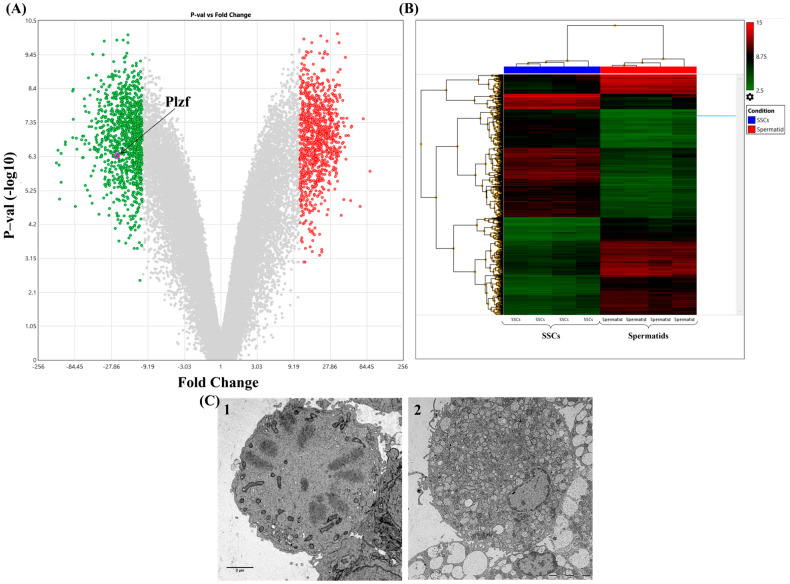
This composite figure presents key SSCs and spermatids. (**A**) The volcano plot shows differentially expressed genes between SSCs and spermatids. Genes with significant upregulation in SSCs are highlighted in green, while those upregulated in spermatids are red. *Plzf*, a key transcription factor in SSC maintenance, is specifically labeled. (**B**) The heatmap illustrates the hierarchical clustering of gene expression across SSC and spermatid samples, with distinct clusters of upregulated and downregulated genes in both cell types. The color scale represents expression levels, from low (green) to high (red). (**C1**,**C2**) Electron microscopy images show the ultrastructure of undifferentiated spermatogonia (**C1**) and differentiating spermatogonia (**C2**), highlighting their morphological differences (Scale bar = 2 µm).

To identify transcriptional changes throughout spermatogenesis, we conducted a differential gene expression analysis comparing spermatogonial stem cells (SSCs) and round spermatids using the Transcriptome Analysis Console (TAC, version 4.0). The analysis utilized CEL files processed on the GPL1261 platform, with normalization performed via the Robust Multi-array Average (RMA) method. Differential expression was assessed with a significance threshold of *p* < 0.05 and a fold change (FC) cutoff of <−2 or >2. For more stringent analysis, genes with a fold change <−11 or >11 were further considered, identifying 1966 differentially expressed genes (DEGs). Among these DEGs, 1010 were downregulated, with *Xlr4*, *Elavl2*, *Mbnl3*, *Cdt1*, *Bex1*, and *Mcm4* being the most significantly downregulated. In contrast, *Slco6d1*, *Hdgfl1*, *Adam1b*, *Dydc1*, and *Slco6b1* were identified as the most highly upregulated among the 956 upregulated genes in round spermatids compared to SSCs (Figure 5). Notably, *Plzf* (*Zbtb16*) was identified as the 243rd most downregulated gene, with a fold change of −23.81 and an FDR *p*-value of 7.03 × 10^−6^ (Supplementary Table S1).

### 3.5. Protein–Protein Interaction (PPI) Networks Construction and Gene Clustering Analysis

**Figure 6:**
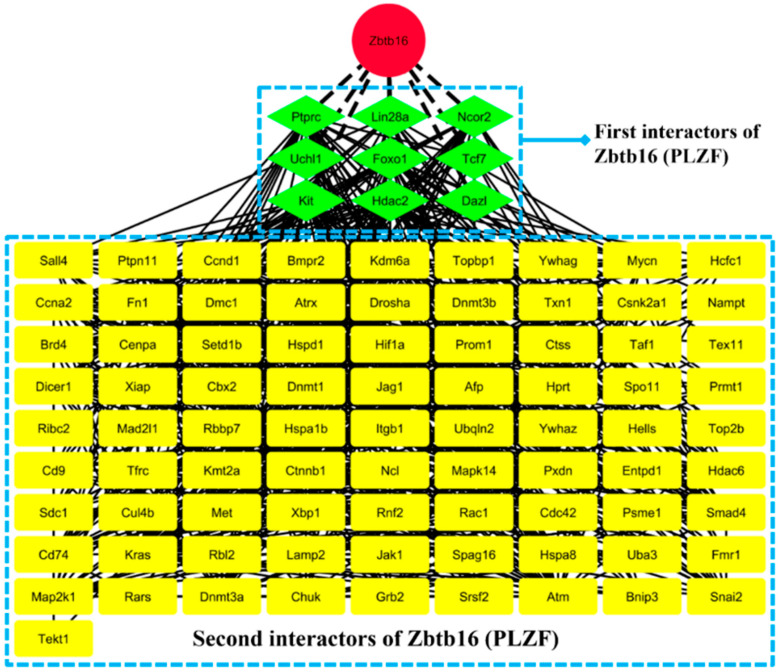
The diagram depicts a protein interaction network for ZBTB16 (PLZF), highlighting its role in spermatogenesis. The red node at the top represents ZBTB16 (PLZF). Direct interactors, indicated by green diamond-shaped nodes, include key proteins such as PTPRC, LIN28A, NCOR2, and others involved in stem cell regulation and differentiation. The yellow rectangular nodes represent the second interactors of ZBTB16, forming an extended network of downstream interactions. The connections suggest a complex.

To construct the protein–protein interaction (PPI) network of key genes identified through microarray analysis, a total of 1966 differentially expressed genes (DEGs) were submitted to the STRING database. The resulting PPI network was subsequently imported into Cytoscape (v. 3.6.0) for further analysis (Supplementary Table S2). To identify the most critical proteins involved in the spermatogenesis process, various network parameters were calculated to assess the importance of each protein. These parameters included degree, betweenness centrality, closeness centrality, and eigenvector centrality. While the first three parameters were computed using Cytoscape’s built-in algorithms, the calculation of the eigenvector centrality required the Centiscape plugin (v. 2.2). After calculating all relevant network parameters, a series of cut-off values were tested to filter and refine the network. Ultimately, a subset of 213 hub genes was identified and deemed crucial for the network (Supplementary Figure S1 and Table S3). This subset of hub genes was then re-submitted to the STRING database to reconstruct a more accurate and reliable PPI network. The process integrates data from multiple sources, including text mining, experiments, databases, co-expression, neighborhood, gene fusion, and co-occurrence information. The newly reconstructed network was then imported into Gephi (v. 0.10.1) to perform modularity-based clustering algorithms. This analysis revealed six distinct clusters within the network, with the Plzf gene emerging as a central node connected to 55 other genes (Supplementary Figure S2 and Table S4). Within this cluster, PLZF interacted with key proteins such as FOXO1, KIT, JAK1, LIN28A, nMYC, KRAS, CD9, AND HIF1. In a protein interaction network, identifying both the primary (first) and secondary (second) partners of a gene like *Zbtb16* (*PLZF*) is essential for uncovering its regulatory role in complex biological processes. Direct interactors, or first partners, are crucial to understanding the immediate functional interactions PLZF engages in, shedding light on its direct influence on cellular pathways. These first interactors can be proteins that physically bind or closely associate with ZBTB16, playing a pivotal role in its immediate biological functions. In contrast, second partners, or indirect interactors, help to reveal the broader signaling cascades and downstream effects that PLZF may modulate, offering insights into its extended influence across multiple pathways. For instance, proteins such as PTPRC, LIN28A, FOXO3, AND NCOR2 might directly interact with PLZF, affecting its functionality, while PLZF, in turn, may directly influence them. Second interactors (indirect partners) such as nMYC, CD9, DNMT1, and SALL4 extend the signaling pathways and provide insight into downstream effects (Figure 6). By observing how the network expands, we can infer how the direct actions of PLZF impact broader cellular processes, forming a cascading effect that might regulate stem cell maintenance or differentiation during spermatogenesis.

### 3.6. Enrichment and Biological Pathways Analysis

**Figure 7:**
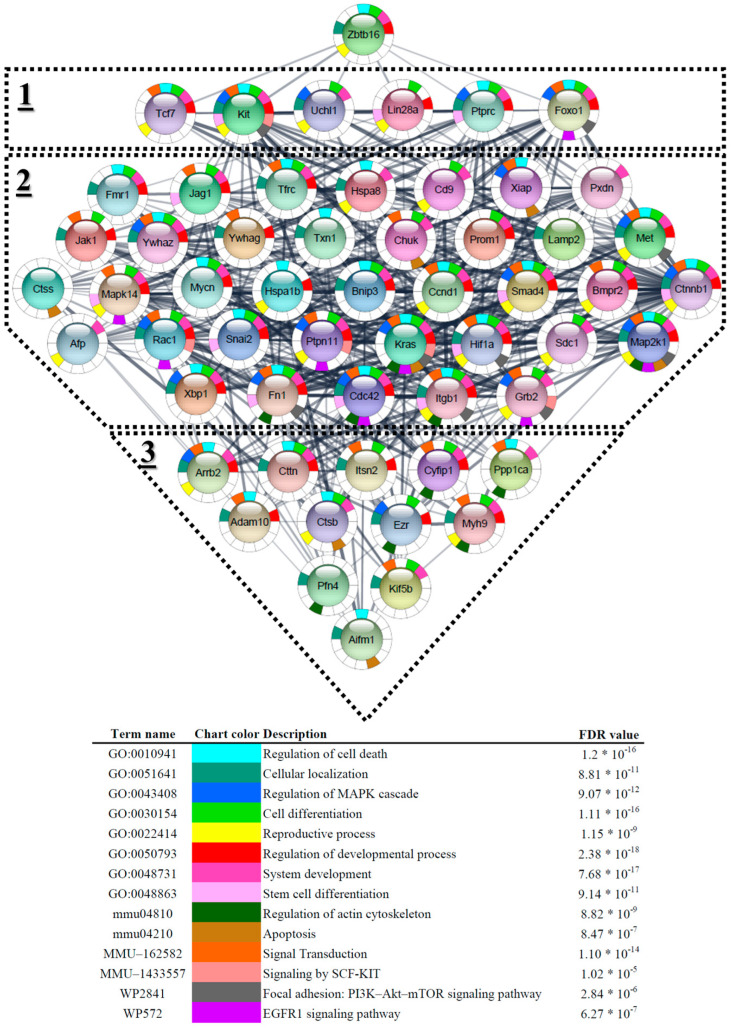
The network diagram illustrates the PPI network centered around ZBTB16 (PLZF), shown at the top, and its hierarchical interactors. The three layers (labeled 1, 2, and 3) represent primary, secondary, and tertiary interactors of ZBTB16, respectively. Each node represents a protein, with the multicolored sectors indicating enrichment analysis results based on the legend below the figure. The edges between nodes indicate predicted interactions between proteins, with a dense network of interconnections suggesting complex regulatory relationships. The color legend at the bottom provides functional context for each protein based on specific biological processes such as signal transduction, chromatin organization, transcription regulation, and others, highlighting the diverse roles of proteins interacting with ZBTB16 in stem cells.

To gain more detailed and accurate insights into the role of the PLZF-associated protein cluster in spermatogenesis, we conducted an independent enrichment analysis and pathway identification of this cluster. The genes within the cluster were imported into the STRING plugin (v. 2.1.1) of the Cytoscape application, with the organism specified as *Mus musculus* (mouse). We then performed enrichment analysis on the constructed protein–protein interaction (PPI) network using Cytoscape, prioritizing the results based on False Discovery Rate (FDR) *p*-values. We investigated Gene Ontology (GO) terms, KEGG pathways, Reactome pathways, and Wikipathways to identify significant PPI networks and the most enriched pathways (Figure 7). Some of the top predicted enriched pathways within the Plzf-associated protein cluster included the “Regulation of MAPK cascade”, “Apoptosis”, “Signaling by SCF-KIT”, “Focal adhesion: PI3K-Akt-mTOR signaling pathway”, and “EGFR1 signaling pathway”. This approach allowed us to highlight the most relevant and statistically significant pathways while also considering less prominent results that could lead to discovering novel and exciting findings.

## 4. Discussion

Spermatogenesis requires an intricate, fine-tuned balance between self-renewal and differentiation of SCC, involving a well-coordinated harmony of organized proliferation, differentiation, and apoptosis in the germ cell niche [19]. The expression of genes associated with stem cell fate determination depends on a multi-phase regulation of intrinsic and extrinsic transcriptional factors and signaling molecules [20]. In the current study, in silico investigation of gene expression microarray datasets identified several differentially expressed genes potentially associated with orchestrating spermatogenesis development. Genes including *Xlr4*, *Elavl2*, *Mbnl3*, *Cdt1*, *Bex1*, and *Mcm4* are among the most significantly downregulated in round spermatids compared to SSCs. Conversely, *Slco6d1*, *Hdgfl1*, *Adam1b*, *Dydc1*, and *Slco6b1* are among the most highly upregulated genes in round spermatids compared to SSCs. *Plzf* is recognized as the 243rd most downregulated gene, with a significant fold change. It underscores the likely indispensable role of PLZF as an intrinsic transcription factor in sustaining the SSC status and lifelong fertility restoration. In line with our reanalysis of the publicly available dataset GSE57197, an in vitro and single-cell sequencing analysis performed by Yang et al. revealed that ELAVL2 is a vital RNA-binding protein that drives the proliferation and survival of human and mouse SSC. Interestingly, their in vitro studies demonstrated that ELAVL2 enhances spermatogonia proliferation by upregulating the ERK and AKT signaling pathways and suppressing SSC apoptosis. Notably, ELAVL2 enhanced the expression of target genes required for SSC self-renewal, including ID4, SALL4, LIN28A, ETV5, and PLZF [21].

Mounting experimental studies have indicated that depletion of *Plzf* in nonsense mutations results in a progressive deficiency in normal spermatogenesis and impaired mature sperm production [22,23]. PLZF, well-known as a multifunctional transcription factor, can function as both a transcription activator and repressor. This indicates its potential capacity to regulate the interplay of intrinsic and extrinsic signals in the maintenance and survival of the SC population [24]. Predicting the possible interaction networks of previously recognized genes is significant in shedding light on the molecular basis of transcriptional changes and intracellular signaling cascades involved in SSC self-renewal and spermatogenesis. While the expression profile of PLZF has been evaluated in most studies, its potential contribution to other genes may provide novel evidence for studies of the SSC population. It is significant to predict the possible interaction networks of previously recognized genes to shed light on the molecular basis of transcriptional changes and intracellular signaling cascades involved in SSC self-renewal and spermatogenesis. As can be seen in the PPI network, KIT, PTPRC, UCHL1, FOXO1, LIN28A, NCOR2, TCF7, DAZL, AND HDAC2 were found to be, potentially, the first key interactors of PLZF in the cluster. Several experimental analyses revealed that PLZF directly suppresses the expression of Kit, a marker for spermatogonia differentiation, to preserve the stemness capacity of SSC [25]. In this regard, Song et al. demonstrated a transcriptional repression function of *Plzf* via binding to the promoter regions of differentiated-related genes, including *Kit*, *Stra8*, *Sohlh2*, and *Dmrt1*, underlining the likely role of *Plzf* as a molecular switch for controlling the Spermatogonia population fate. Additionally, they observed that *Kit*, *Stra8*, *Sohlh2*, and *Dmrt1* were highly expressed in *Plzf* knockdown Spermatogonia cells [13]. FOXO1 can influence SSC hemostasis and differentiation initiation by directly and indirectly regulating the genes expressed in SSC. Mice with Foxo1 depletion displayed similar defects in SSC self-renewal and survival to the maintenance genes, including Plzf, Taf4b, and Etv5 [26]. Intriguingly, Shen et al. highlighted that the expression level of stemness-related genes, including *Plzf* and *Oct4*, decreased in *Foxo1*-depleted SSC, and the *Kit* expression (differentiation marker) was upregulated [27]. It has been suggested that PLZF directly and indirectly (via, e.g., FOXO1 and ETV5) may suppress differentiation-associated genes (including *Kit*) [28]. Thus, it may be concluded that FOXO1 and PLZF displayed a complex interconnected association to preserve a coordinated balance in stemness, differentiation, and cell cycle. LIN28A, an RNA-biding stem cell factor, downregulates let-7 microRNA, contributing to the pluripotency, self-renewal, and stemness capacity [29]. LIN28 and OCT4, SOX2, and NANOG expression can induce pluripotent stem cells from human somatic cells [30]. In the setting of SSC, *Lin28* was shown to be markedly expressed in undifferentiated spermatogonia (As, Apr, and Aal) in mice [31,32]. Interestingly, Ma et al. found that LIN28A preserved the self-renewal of dairy goats by enhancing the expression of *Oct4*, *Sox2*, *Gfra1*, *Plzf*, and *Etv* in vitro and in vivo [33]. On this point, glial-cell-derived neurotrophic factor (*GDNF*), expressed by Sertoli cells, binds to the cRET/GFRa1 receptor in SSC and induces the transcriptional upregulation of *Lin28a* and *Plzf* to conserve the stemness state of spermatogonia reservoirs [34]. As the PPI network suggested that PLZF connects with LIN28A through the first interaction, it indicates that PLZF and LIN28A interconnection play an essential role in SSC pool maintenance.

As can be noticed in the PLZF interactions network, UCHL1 has been proposed as one of the PLZF partners. The precise expression level of UCHL1 is highly associated with SSC survival and differentiation in the spermatogonia population expressing *Plzf* [35]. Lou et al. found that *Uchl1* exhibited a high expression in spermatogonia with positive *Plzf* expression located at the basement membrane and conversely had low or absent expression in differentiated populations with positive DAZL, DDX4, and c-KIT markers. Of special consideration, during the asymmetric division of germline stem cells, *Uchl1* and *Plzf* expression is sustained strongly in spermatogonia at the basement membrane. However, its expression is diminished or absent in daughter cells away from the basement membrane. Their result highlighted that asymmetric segregation of UCHL1 and PLZF may particularly impact SSC fate decisions [36]. Surprisingly, both UCHL1 and PLZF might play a potential synergistic role in negatively regulating mTORC1 pathway activity, all of which could maintain the capacity of SSC to self-renew [35,37,38]. For instance, a study reported that spermatogonial progenitor cells with *Plzf* mutation showed a remarkable upregulation in the mTORC1 pathway, which impeded their response to GDNF and the self-renewal of spermatogonia reservoir [38]. In other words, investigators have postulated that PLZF opposed the regulation of mTORC1 by upregulating the mTORC1 inhibitor REDD1, preserving the effect of GDNF on stemness maintenance [39]. With regards to the crucial role of the appropriate activity of mTORC1 in spermatogonial development, Wang et al. observed an noticeable decrease in PLZFs’ mRNA and an increase in STRA8s’ and C-KITs’ mRNA in mutated mice with aberrant expression of mTORC1 pathway, leading to impaired spermatogenesis and subfertility [40]. Therefore, we propose that there may be a multi-phase interaction between PLZF, UCHL1, mTORC1, and GDNF signaling in regulating the self-renewal and differentiation capacity of the SSC population. SALL4 appeared to be involved in the second interaction with PLZF in the PPI network. The interaction between PLZF and SALL4 transcription factors in the literature ensures the proper sustainability of undifferentiated states and self-renewal properties [41]. ChIP-seq in mice SSC in a study performed by Lovelace et al. demonstrated that PLZF and SALL4 coordinately bind to promoters or introns of target genes, such as those involved in coordinate regulation of SSC survival and differentiation. In other words, PLZF and SALL4 co-bound genomic zones include PLZF binding sites, highlighting that PLZF engages SALL4 to the target genes. It has been suggested that the PLZF-SALL4 complex might enhance self-renewal by activating the genes associated with spermatogonia proliferation or repressing the differentiation genes [42]. However, Chan et al. pointed toward a PLZF-independent function of SALL4 in the spermatogonia pool [43]. The possible connection of PLZF with its related proteins in the PPI network cluster necessitates further fundamental research efforts to unravel the underlying processes within this pathway.

Based on our in silico enrichment analysis, the PLZF-associated protein cluster is predicted to be recruited in key pathways including the “Regulation of MAPK cascade”, “Apoptosis”, “Signaling by SCF-KIT”, “Focal adhesion: PI3K-AKT-mTOR signaling pathway”, and “EGFR1 signaling pathway”. MAPK pathway is essential in governing cell cycle progression and differentiation and can be markedly upregulated in cancerous mice models and patient-derived tumor samples [44]. Although the direct interaction between PLZF and MAPK signaling pathway has not been investigated in SSC, much evidence from prostate cancer research uncovered the potential connection between PLZF and its connected pathway. For instance, Bioinformatic analysis in a study by Hsieh et al. in prostate cancer cells suggested that PLZF negatively regulates the MAPK pathway via repressing ERK1/2 signaling to prevent sustained cancer cell growth [45]. Hence, it may be assumed that PLZF regulates the MAPK/ERK downstream signals to ensure the maintenance of the intracellular signaling at a level that safeguards the balance between SSC self-renewal and aberrant proliferation. The PI3K-AKT-mTOR signaling pathway coordinates spermatogonia proliferation, differentiation, and apoptosis [46]. Jia et al. demonstrated that AKT3 contributed to the SCC self-renewal, proliferation, and differentiation through the PI3K-AKT-mTOR pathway [47]. PTEN, a negative regulator of PI3K-dependent AKT signaling, has been pointed to control the fate of SSC by regulating the PLZF signaling [48]. Interestingly, Zhou et al. reported that Pten-deleted SSCs were associated with decreased expression of *Plzf*, gradually resulting in SSC homeostasis impairment and infertility. Moreover, the authors found that *Pten* knockout mice underwent overgrowth of testes with unstable premature large cells [49]. Thus, it is likely to assume that the downregulation in *Pten* is associated with the overdrive PI3K-AKT-mTOR pathway, depletion of *Plzf*, excessive proliferation of SSC, and disturbed SSC pool.

Consistent with the microarray analysis, the findings from the Fluidigm qPCR indicated a significant expression of Plzf mRNA in basal compartment cells, which contain undifferentiated spermatogonia, compared to the differentiated spermatogonia. Similar to our experimental analysis, Azizi et al. found that *Plzf* was highly expressed in OCT4+/VASA- undifferentiated spermatogonia in the basal compartment of seminiferous tubules in adult testis, but neither in the differentiating germ cells [48]. Immunostaining studies further revealed results similar to those of the Fluidigm analysis, suggesting the pivotal role of *Plzf* expression in undifferentiated spermatogonia. In addition, immunohistochemistry showed that PLZF-positive undifferentiated spermatogonia minimally expressed *nMyc* and *Vasa*, and their expression was significant in germ cells located in the middle compartment of the seminiferous tubules. Previous studies confirmed that VASA may be a germline differentiation marker with evident expression in spermatocyte and round spermatid, but neither in PLZF-positive SSC [50]. However, Braydich-Stolle et al. unraveled that the GDNF-GFRα-1-Ret pathway upregulates the expression of *nMyc* through SRC and PI3K activation, possibly leading to the proliferation and maintenance of SSC reservoir [51].

Our experimental analysis confirmed the significant expression of Plzf in undifferentiated spermatogonia, albeit it may not simply reflect the same observation in human spermatogonia. Hermann et al. performed a comprehensive single-cell transcriptome of spermatogenic cells, which highlighted considerable gene expression heterogeneity among adult spermatogonia in mice and humans. Notably, the expression pattern of PLZF in human spermatogenic clusters through meiosis was found to be unique to humans [52]. With the significant distinctions between rodent and human spermatogenesis, there has been a growing interest in diving deeper into the spermatogenesis process in humans. While specific biomarkers such as ENO2, LIN28, PLZF, SALL4, SSEA4, UCHL1, and UTF1 primarily label undifferentiated spermatogonia, emerging evidence indicates that they also mark differentiating spermatogonia in humans [53,54]. The single-cell RNA-sequencing analysis of human testis demonstrated that human ID4 and PLZF were broadly enriched for spermatogonia subsets, not only the undifferentiated human spermatogonia [55]. Similarly, another study revealed that regardless of PLZF expression in undifferentiated As, Apr, and Aal spermatogonia, its expression overlapped with early differentiating spermatogonia in humans [54]. Thus, the possible selectivity of PLZF expression for SSC is likely dependent on the studied species.

In the current study, we presented both in silico analysis and in vitro experiments, highlighting the potential role of the PLZF transcription factor in coordinating the balance between differentiation and self-renewal in the SSC population. Based on the PPI network, PLZF may display an intricate interaction with possible proteins, such as NMYC, LIN28A, FOXO1, AND UCHL1, to preserve the high-throughput and life-long spermatogenesis. Unraveling the cellular and molecular mechanisms involved in SSC self-renewal and differentiation signaling pathways plays a fundamental role in regenerative medicine and cell-based therapies.

## 5. Conclusions

Spermatogonial stem cells are the foundation of continuous spermatogenesis and productive fertility, underscoring their clinical application in reproductive disorders. Progress in understanding the underlying molecular and signaling pathways of SSC regulation and proliferation promotes novel strategies for clinical intervention. In this study, we elucidated the potential role of PLZF in preserving the undifferentiated state of spermatogonia by performing in silico and experimental analysis. Based on the in vitro analysis, the predominant expression of Plzf in undifferentiated spermatogonia highlighted its crucial role in maintaining SSC restoration. PLZF may represent an interaction with proteins, including NMYC, LIN28A, FOXO1, JAK1, AND UCHL1 to enrich pathways associated with SSC self-renewal and differentiation possibly. Identifying further signaling pathways and transcriptional changes associated with PLZF paves the way for developing a promising avenue for research and clinical applications in the context of reproductive and oncology medicine.

## Data Availability

The data presented in this study are available in NCBI GEO at https://www.ncbi.nlm.nih.gov/geo/ (accessed on 18 November 2024), accession number GSE57197, and reference number [17].

## Supplementary Materials

The following supporting information can be downloaded at: https://www.mdpi.com/article/10.3390/cells13231930/s1.
