# Supplementary material for: The Role of Plzf in Spermatogonial Stem Cell Maintenance and Differentiation: Mapping the Transcriptional Dynamics and Key Interactions
Source: Cells. 2024 Nov 21;13(23):1930. doi: 10.3390/cells13231930 (PMC11640652; doi:10.3390/cells13231930)
Supplement: Supplementary file 1 [file cells-13-01930-s001.zip › Supplementary.pdf]

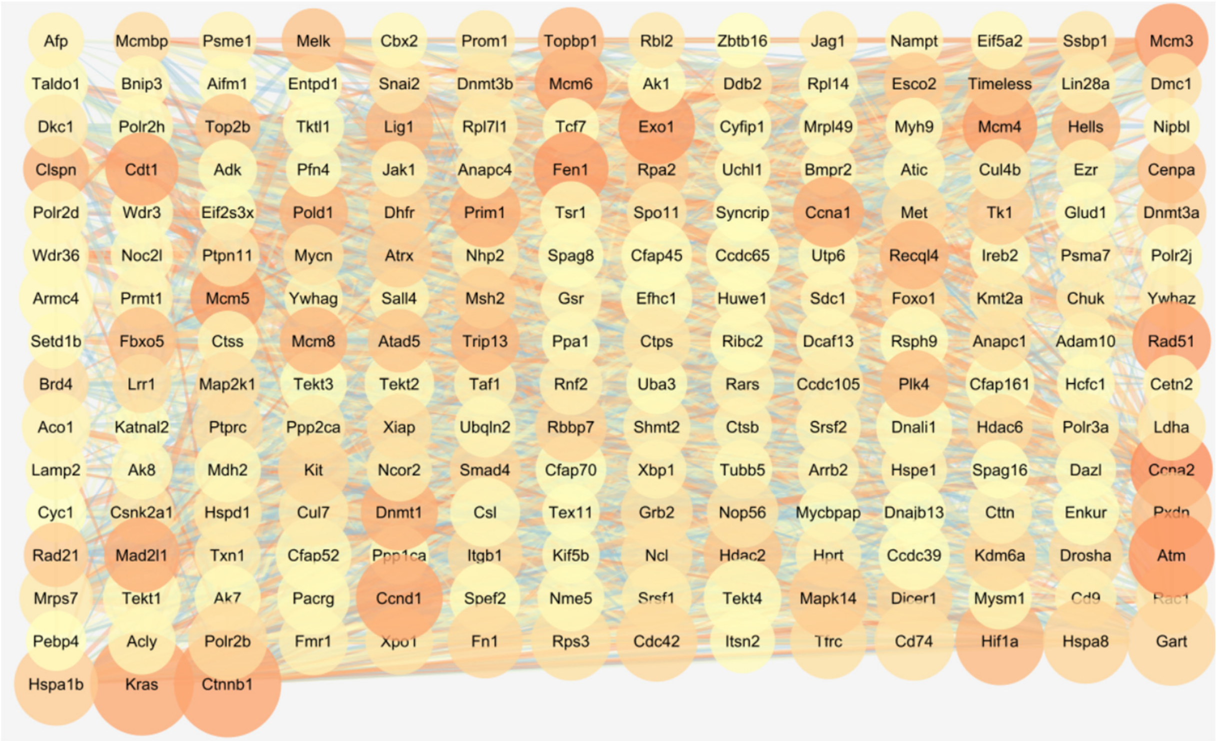

Figure S1. The network visualization represents gene co-expression patterns, where each node corresponds to a gene, and the connecting edges represent the strength of gene-gene co-expression interactions. The size of the nodes reflects the degree, with larger nodes indicating genes with a higher number of connections. The darker-shaded nodes signify higher eigenvector centrality, emphasizing genes that are more central and influential within the network. Edge thickness corresponds to the co-expression strength, with thicker edges representing stronger correlations between gene pairs. Additionally, the darker edges indicate higher combined scores, highlighting more biologically significant interactions.

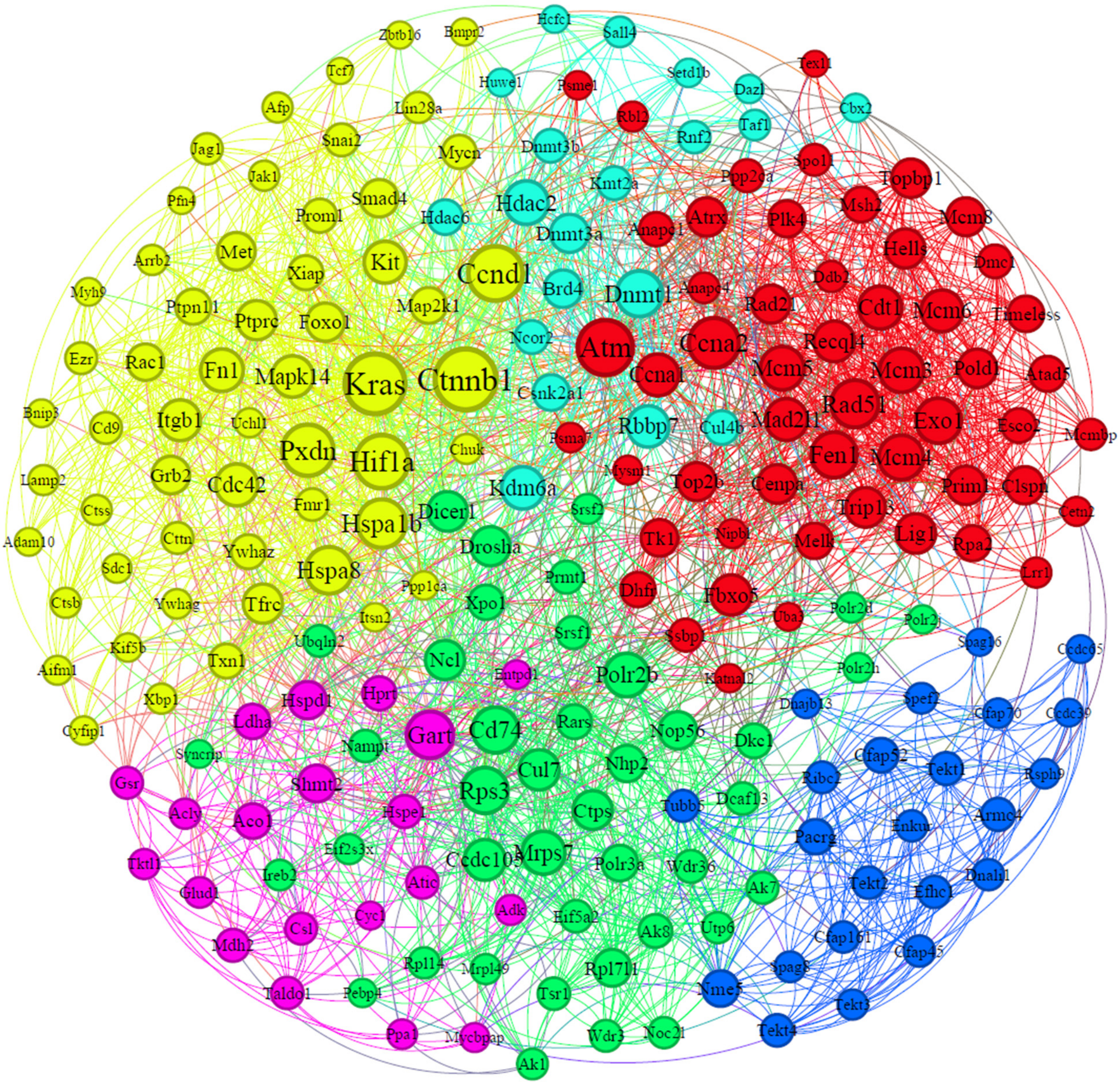

Figure S2. A total of six clusters in six distinct colors were specified with a modularity algorithm and visualized in a PPI network by Gephi. Larger nodes have more connected nodes and larger degrees.
